# Lactic Acid Permeation through Deep Eutectic Solvents-Based Polymer Inclusion Membranes

**DOI:** 10.3390/membranes10090244

**Published:** 2020-09-19

**Authors:** Michiaki Matsumoto, Sae Takemori, Yoshiro Tahara

**Affiliations:** Department of Chemical Engineering and Materials Science, Doshisha University, Kyoto 6100321, Japan; tknkxxsae@gmail.com (S.T.); ytahara@mail.doshisha.ac.jp (Y.T.)

**Keywords:** polymer inclusion membrane, deep eutectic solvent, lactic acid

## Abstract

Lactic acid that is prepared by fermentation is a compound in food, cosmetic pharmaceutical, and chemical industries. Since a simple technique is desired that separates lactic acid from the cultures, we propose lactic acid permeation through a poly(vinyl chloride)(PVC)-based membrane that contains deep eutectic solvents (DESs) as a carrier. Lactic acid was successfully permeated through polymer inclusion membranes (PIMs) containing hydrophilic DESs, urea-choline chloride and glucose-choline chloride. The permeation behavior was explained by the facilitated transport mechanism based on the solution-diffusion model. Simple preparation of thinner membranes in the PIM process and higher permeation rates are advantages over the supported liquid membrane process. The PVC-based membrane process containing environmentally benign hydrophilic DESs is promising for lactic acid separation on an industrial scale.

## 1. Introduction

Lactic acid, which is prepared by fermentation, is a critical compound in food, cosmetic pharmaceutical, and chemical industries [1]. In the fermentative production processes of lactic acid, 40–70% of the operating and capital costs are associated with the separation steps [2]. A chemical precipitation method has been industrially applied as a separation technique [3]. A simple and economical technique is required that separates lactic acid from the fermentation broth. The proposed separation techniques involve solvent extraction, reactive distillation, electrodialysis, aqueous two-phase systems, ion exchange, adsorption, and liquid membranes [3,4,5]. In our previous studies, we proposed supported liquid membranes (SLMs) and polymer inclusion membranes (PIMs) based on ionic liquids (ILs), such as Aliquat 336 and Cyphos IL-101 for lactic acid separation [6,7,8], and lactate successfully permeated through both membranes. Recently, deep eutectic solvents (DES) emerged as a new generation of ILs prepared by mixing a hydrogen bond acceptor (HBA) and a hydrogen bond donor (HBD) with remarkably low volatility [9]. When DES was prepared from such naturally occurring HBAs as choline chloride and HBDs as urea and glucose, the prepared DESs were environmentally benign, and sustainable. Therefore, DESs have been used as a membrane liquid for the SLM process in the separation of olefin [10,11,12], CO_2_ [13,14], antiarrhythmic agents [15], furfural [16], and amino acids [17]. In the separation of furfural and hydroxymethylfurfural, hydrophobic DESs and supporting membranes were examined, and a DES that consisted of thymol and lidocaine, which was impregnated in a polyethylene support, enhanced permeability [16]. On the other hand, in the separation of amino acids, the hydrophilicity of DESs and the membranes is important for permeability and stability. A DES consisting of choline chloride and *p*-toluenesulfonic acid (PTS) was the most efficient [17]. In the separation of antiarrhythmic agents, relatively hydrophobic DESs were studied, and a DES consisting of choline chloride and 1-phenylethanol was selected as an effective, green membrane liquid [15]. A common feature shared by previous papers [15,16,17] is that no carriers were used for the permeable compounds. Because amino acids are amphoteric, DES also played the role of transport carrier.

In this study, we applied PIMs containing DESs to the separation of lactic acid. PIMs are formed by casting a solution that contains a carrier, a plasticizer, and a base polymer such as cellulose triacetate (CTA), poly(vinyl chloride)(PVC), or poly(vinylidene fluoride-co-hexafluoropropylene) (PVDF-HFP) to form a thin, flexible, and stable film. Their superior stability over SLMs has been reported [8].

## 2. Materials and Methods

### 2.1. Chemicals

The base polymers we used were polyvinyl chloride (PVC) from Fujifilm Wako Chemicals, Osaka (Japan). (*n* = 1100) and poly(vinylidene fluoride-co-hexafluoropropylene) (PVDF-HFP) from Sigma-Aldrich, St. Louise (MO, USA). Because CTA is susceptible to hydrolysis in acidic and alkali media [18], we used PVC and PVDF-HFP as the base polymers. Lactic acid was also purchased from Fujifilm Wako Chemicals, and diluted with ion-exchanged water to approximately 1.0 mol/dm^3^ and heated for at least 12 h to hydrolyze the lactic acid polymers. The concentrated hydrochloric acid and concentrated sodium hydroxide solutions (Fujifilm Wako Chemicals, Osaka, Japan) were used for adjusting the pH of the receiving phases. All the chemicals including HBA and HBD (Fujifilm Wako Chemicals, Osaka, Japan) were used without further purification.

### 2.2. DES Preparation

We prepared the DES by mixing HBD and HBA [19] listed in Table 1 by the following procedure according to a previous paper [20]. Table 1 also shows their hydrophobicity expressed by Log *K*_O/W_(HBD) + Log *K*_O/W_(HBA). The mixture was stirred at 80 °C until a clear homogenous mixture was formed. Before being used, the DESs were left overnight at room temperature to confirm whether recrystallization had occurred. We selected the DESs with a wide range of log *P* values listed in Table 1. In the present study, we used PIMs containing hydrophilic and hydrophobic DESs as a lactic acid carrier without a plasticizer, because DESs are expected to play carrier and plasticizer roles.

### 2.3. Membrane Preparation

**Figure 1:**
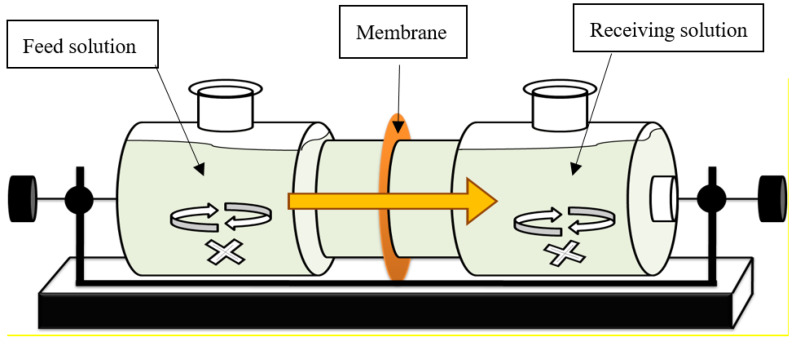
Experimental apparatus for permeation of lactic acid through a membrane.

According to a previous procedure [8], the PIM was prepared by a solution casting method. We proposed a polymer solution by dissolving the PVC (0.6 g) and the DES (1.5 g) in 25 mL tetrahydrofuran or cyclohexanone. The solution mixture was stirred with a homogenizer and poured into a flat plate dish. After evaporating the solvent for 24 h at room temperature, we obtained a typical resultant PIM with an average film thickness of 280 μm using a micrometer (Digimatic, Mitsutoyo, Kawasaki, Japan). The PIM was a flexible and stable film without a specific plasticizer. The polymer membranes had an effective area, *A*, of 12 cm^2^ and were fixed in the apparatus as shown in Figure 1. For the PVDF-HFP-based PIM, N,N-dimethylacetoamide was used as a solvent.

To compare PIM with SLM, the permeation of lactic acid through an SLM that consisted of DES was carried out. We used hydrophilic or hydrophobic porous polyvinylidine fluoride (PVDF) membranes (Millipore Corporation, Billerica, MA, USA) as support to maintain the DES in the membrane, whose thickness was 125 µm, and whose pore size was 0.45 µm. A hydrophilic or hydrophobic PVDF was soaked in hydrophilic or hydrophobic DES in a flat plate dish. The membrane was left in the soaked state for 24 h at room temperature, wiped, and dried in vacuum desiccators for 24 h.

### 2.4. Permeation Experiment

The apparatus shown in Figure 1 for our permeation experiment consists of feed and receiving phases. The feed solution was a lactic acid (0.1 mol/m^3^) aqueous solution without a pH adjustment. The receiving solution was 0.1 mol/dm^3^ sodium hydroxide solution. In most SLM and PIM studies, the concentration of permeable compounds is within the range of a few milli-molarity. In the present study, we used a lactic acid concentration of 0.1 mol/dm^3^ to obtain a closer lactic acid concentration in the broth. To examine the pH effect in the receiving phase, the pH was adjusted using a hydrochloric acid or a sodium hydroxide solution. Each compartment was filled with 100 mL of the respective solution at room temperature under the initiated conditions. The stirring speed of the magnetic bar in each cell was controlled at 300 rpm. The permeation experiment was carried out for 48 h. Samples from both solutions were withdrawn at regular intervals, and the pH in both cells was measured with a pH meter. Lactic acid concentrations were determined using an HPLC (LC-20 AD, Shimadzu) with a refractive index detector (RID-10 A, Shimadzu) with a Shodex SUGAR SH-1011 (Showa Denko K. K.) column and 5 mmol/dm^3^ sulfuric acid solution as the mobile phase.

## 3. Results and Discussion

**Figure 2:**
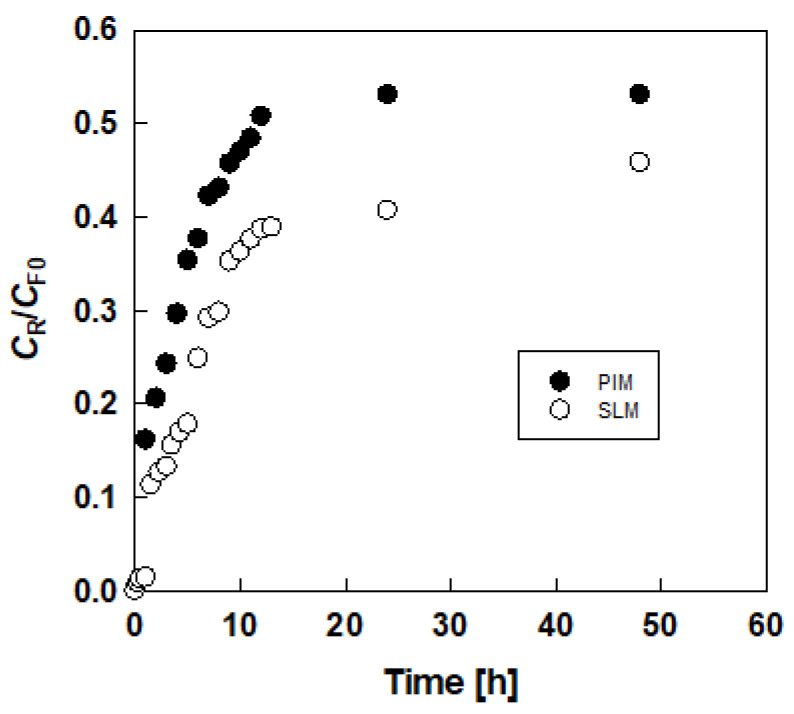
Typical results of permeation of lactic acid through polymer inclusion membranes (PIM) and supported liquid membranes (SLM).

Figure 2 shows the typical time-courses of the dimensionless lactic acid concentration in the receiving solution (*C*_R_/*C*_F0_) using a DES that consists of choline chloride and glucose in PIM and SLM. The lactic acid concentration gradually increased with elapsed time (*t*) and reached plateau region (*C*_R∞_). The permeation rate in PIM was higher than that of SLM.

Assuming that the permeation rate is driven by deviation from the plateau concentration, the following equation can be written.

(1) $$-\frac{dC_{F}}{dt}=\frac{dC_{R}}{dt}=P\left(C_{R\infty }-C_{R}\right)$$

where *C* is the lactic acid concentration (mol/dm^3^), *P* is the apparent permeability (h^−1^), and subscripts F and R denote the feed and receiving solution. Integrating Equation (1) with initial condition *C*_R_ = 0 at *t* = 0 gives:

(2) $$\ln\left(\frac{C_{R\infty }}{C_{R\infty }-C_{R}}\right)=Pt$$

**Figure 3:**
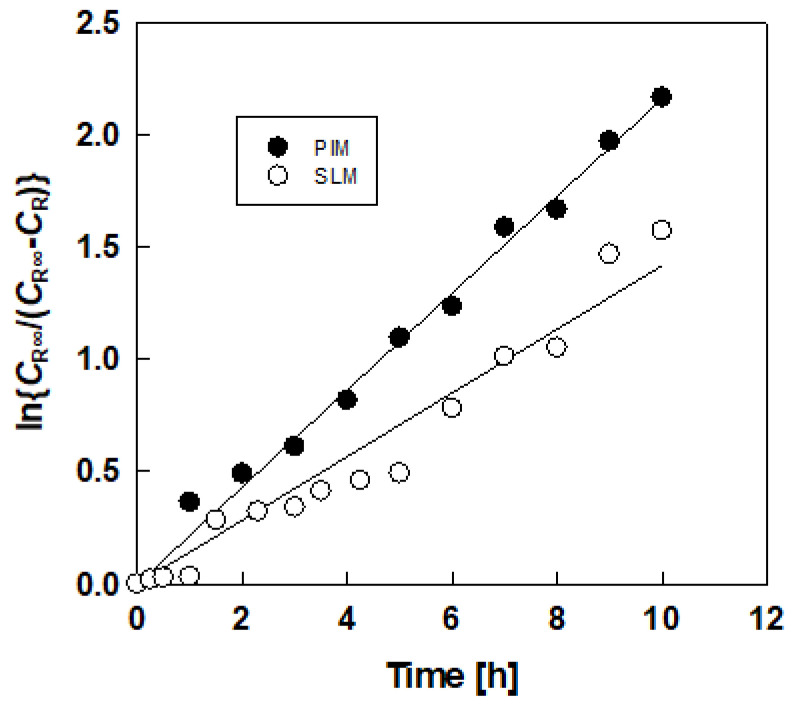
Typical results of determination of apparent permeability from plots based on Equation (2).

Figure 3 shows the relations based on Equation (2) in the initial stage, and linear relationships between $\ln\left(\frac{C_{R\infty }}{C_{R\infty }-C_{R}}\right)$ and *t* were obtained, suggesting that assumption of Equation (1) is valid. The *P* values were calculated from the slopes of the straight lines.

In this paper, we evaluated two parameters, *P* and *C*_R_/*C*_F0_, at 48 h, for the membrane performance.

### 3.1. Effects of DES and Membrane

**Figure 4:**
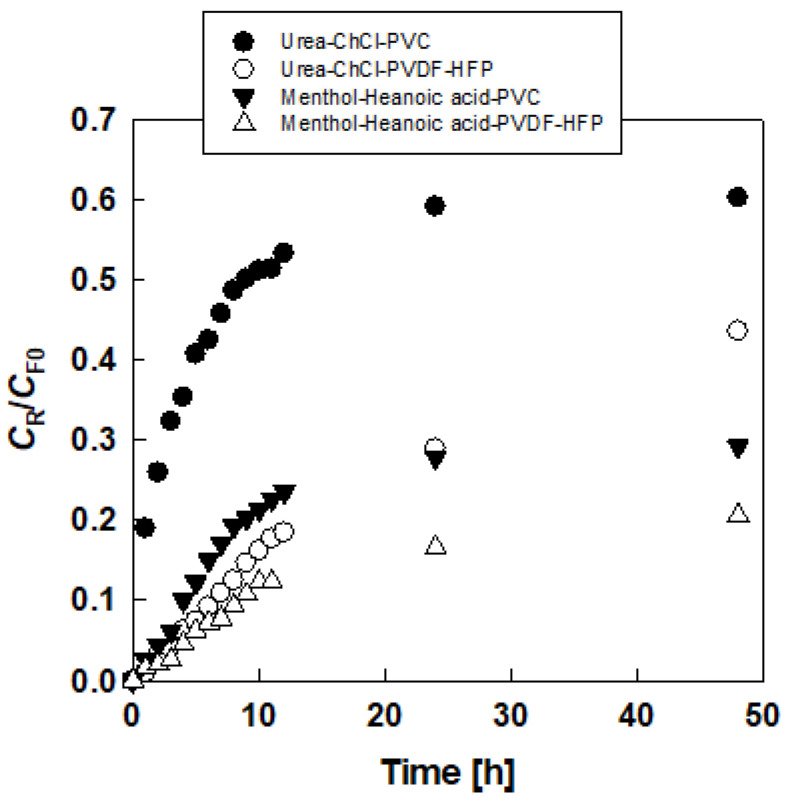
Effect of base polymer on permeation of lactic acid through DES-based PIM.

Table 2 shows the effects of DESs on *P* and *C*_R48_/*C*_F0_. More hydrophilic DESs, urea-choline chloride and glucose-choline chloride evidently, gave larger values of both *P* and *C*_R_/*C*_F0_. It was found that although a difference in *P* values was relatively small, *C*_R48_/*C*_F0_ values greatly depended on the hydrophilicity of DESs. PVDF-HFP-based PIM recently showed better permeability and membrane stability than a PVC-based PIM containing a chelating extractant for metal permeation [20]. For urea-choline chloride and DL-menthol-hexanoic acid DESs, lactic acid permeations through PVDF-HFP-based PIM were examined as shown in Figure 4. In both DESs, PVC-based PIM gave higher lactic acid permeation rates. This identified an opposite trend from a previous PIM study [21]. However, in amino acid permeation through DES-based SLM [17], the consistent hydrophilicity of membranes and DESs is important for excellent membrane performance. Since the water contact angles for PVC [22] and PVDF-HFP [23] were 83° and 95.5°, respectively, PVC is more hydrophilic. Therefore, in a DES-based PIM, both the membrane and DES are hydrophilic. Urea, glucose, and choline chloride are preferred due to their low cost and nontoxic natures. Although the interaction of the membrane and DES probably contributes to the membrane performance, elucidating this idea remains difficult. This will be clarified in our next step.

In a previous paper on DES-based SLM [17], the initial concentration range of a permeable compound, tryptophan (Trp), was 0.1–0.5 mM, and its extraction efficiency decreased with increasing Trp concentration and was less than 50% at an 0.5 mM Trp concentration. In this case, even if a very high initial concentration (0.1 M) was used, we obtained a high extraction efficiency over 60%. 

### 3.2. Permeation Mechanism

**Figure 5:**
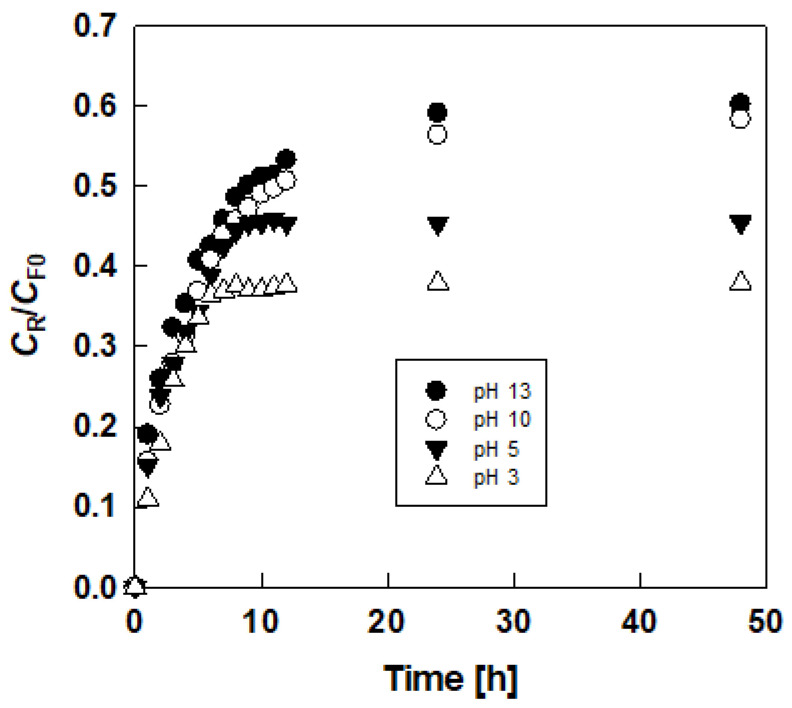
Effect of pH of receiving solution on permeation of lactic acid through urea-choline chloride-based PIM.

To discuss the permeation mechanism, we examined the pH effect of the receiving solution on the permeation as shown in Figure 5. A pH increase in the receiving solution caused a high permeation rate. Since the pH of the feed solution was 2.5 without a pH adjustment and the pKa of the lactic acid was 3.86, the undissociated state of the lactic acid in the feed solution and the dissociated state in the receiving solution were preferred for the permeation. Therefore, the undissociated lactic acid in the feed solution was distributed to the membrane phase and diffused in the membrane. A stripping reaction at the interface between the membrane and receiving phases ocurred. When the pH of the receiving solution was high, lactic acid distributed to the receiving phase was converted to its dissociated form. Therefore, permeation was facilitated by the alkaline receiving solution.

**Figure 6:**
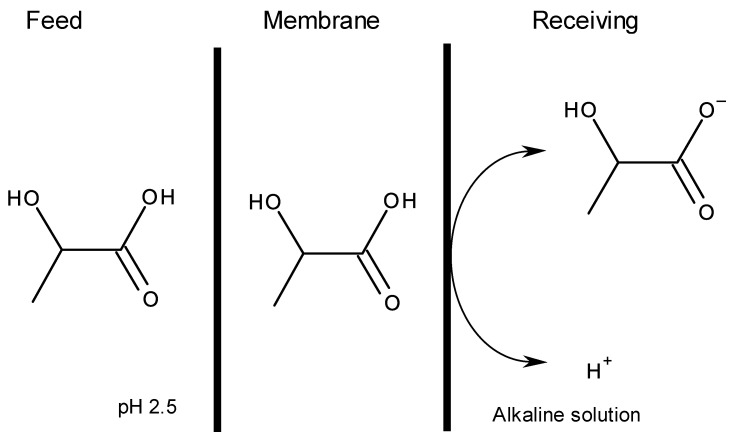
Facilitated transport of lactic acid DES-based PIM.

In our previous paper on lactic acid permeation through ionic liquid-based PIM [8], permeation followed the solution-iffusion mechansm and was controlled by the solution step. In this study, we did not examine the distribution behavior of lactic acid between the DESs and water because some DESs are water soluble. However, water-soluble DESs in a PIM played a role of the transport carrier, suggesting interaction between the membrane and the DESs. Figure 6 schematically represents the permeation mechanism.

### 3.3. Comparing PIM with SLM

Figure 2 and Table 2 also compare a PIM with a SLM that contains DES. For the hydrophobic DES-based SLM, the lactic acid did not permeate at all (data not shown in Table 2). This behavior resembles amino acid permeation through a DES-based SLM [17]. Even though the membrane of PIM is thicker than that of SLM, PIM gave a higher permeation rate than SLM. As shown in Figure 4, PVDF-co-HFP membrane gave lower permeation than PVC in PIM. This result also applies to the SLM of this study. 

## 4. Conclusions

We conducted the permeation of lactic acid through a PVC-based membrane containing DESs as a carrier. Lactic acid was successfully permeated through PIMs containing hydrophilic DESs, urea-choline chloride and glucose-choline chloride. Hydrophobic DESs were unsuitable as a membrane carrier for PIMs because of a low permeation rate. The permeation behavior was explained by a facilitated transport mechanism based on the solution-diffusion model. Simple preparation of thinner membranes in the PIM process and higher permeation rates are advantages over the SLM process. The PVC-based membrane process containing environmentally benign hydrophilic DESs is promising for lactic acid separation.

## Figures and Tables

**Table 1:** Deep eutectic solvents (DES) in our work and previous studies.

No	HBD	HBA	HBD:HBA (Molar Ratio) [19]	Log *K*_O/W_(HBD) + Log *K*_O/W_(HBA) (*1)	Ref.
1	Urea	Choline chloride	2:1	−7.7	
2	Glucose	Choline chloride	1:2	−8.3	
3	Decanoic acid	Tetrabutylammonium chloride	2:1	4.9	
4	Octanoic acid	Tetrabutylammonium chloride	2:1	3.9	
5	Hexanoic acid	Tetrabutylammonium chloride	2:1	2.8	
6	Octanoic acid	Tetraethylammonium chloride	2:1	−0.1	
7	Hexanoic acid	Tetraethylammonium chloride	2:1	−1.2	
8	Octanoic acid	Lidocaine	2:1	5.3	
9	Hexanoic acid	Lidocaine	2:1	4.2	
10	Octanoic acid	DL-Menthol	2:1	6.4	
11	Hexanoic acid	DL-Menthol	2:1	5.2	
12	Thymol	Lidocaine	2:1	−1.4	16
13	PTS	Choline chloride	2:1	−5.6	17
14	1-Phenylethanol	Choline chloride	4:1	−3.3	15

(*1) *K*_O/W_ is the octanol-water partition coefficient.

**Table 2:** Permeation characteristics of DES.

DES (*1)	*P* (h^−1^)	*C*_R_/*C*_F0_ at 48 h
1	0.204	0.60
1S (*2)	0.146	0.48
2	0.216	0.53
2S (*2)	0.142	0.46
3	0	0.06
4	0.175	0.07
5	0.099	0.11
6	0.167	0.10
7	0.128	0.16
8	0.091	0.16
9	0.098	0.20
10	0.103	0.26
11	0.125	0.29

(*1) DES numbers correspond to those in Table 1. (*2) S denotes the SLM using PVDF. Others are the results of PVC-based PIM.
